# Cost-effectiveness analysis of selexipag for the combined treatment of pulmonary arterial hypertension

**DOI:** 10.3389/fphar.2023.1122866

**Published:** 2023-08-11

**Authors:** Wenxing Dong, Zhe Zhang, Mingming Chu, Peng Gu, Min Hu, Lulu Liu, Jingbin Huang, Rong Zhang

**Affiliations:** ^1^ Department of Pharmacy, The Second Affiliated Hospital of Army Medical University, Chongqing, China; ^2^ Department of Pharmacy, Beidaihe Rehabilitation and Recuperation Center of Joint Logistics Support Forces, Qinhuangdao, China

**Keywords:** cost-effectiveness, markov model, selexipag, pulmonary arterial hypertension, endothelin receptor antagonists, phosphodiesterase 5 inhibitor

## Abstract

**Objective:** Adding selexipag to the combined treatment of endothelin receptor antagonists (ERA) and phosphodiesterase 5 inhibitor (PDE5i) reduces the risk of clinical worsening events in patients with pulmonary arterial hypertension (PAH) but at a considerably higher cost. This study evaluated the cost-effectiveness of adding selexipag to the combined treatment of ERA and PDE5i in patients with PAH from a Chinese healthcare system perspective.

**Methods:** A Markov model was developed to assess costs and quality-adjusted life years (QALYs) of macitentan + tadalafil + selexipag vs. macitentan + tadalafil for the treatment of PAH. Markov states included WHO Functional Class (FC) (I–IV) and death. Transition probabilities were based on data from the TRITON trial. Mortality rates, costs, and utilities were obtained from published literature and public databases.

**Results:** In the base case analysis, compared with macitentan + tadalafil, selexipag + macitentan + tadalafil increased costs ($357,807.588 vs. $116,534.543, respectively) and QALYs (7.234 QALYs vs. 6.666 QALYs, respectively). The resulting incremental cost-effectiveness ratio was $424,746.070 per QALY, which was higher than the willingness-to-pay (WTP) of $38,223.339 per QALY. The results were most sensitive to HR for mortality of patients with FC IV relative to the general population, discount rate, and the cost of selexipag. The probability was greater than 50% for the selexipag + macitentan + tadalafil only if the WTP was more significant than $426,019.200 per QALY.

**Conclusion:** In China, adding selexipag may not be cost-effective for patients with PAH who failed to control their condition after combined treatment of ERA and PDE5i. Results of the analysis can aid discussions on the value and position of selexipag for the combined treatment of PAH.

## 1 Introduction

Pulmonary arterial hypertension (PAH, group 1) is a rare, progressive, and fatal disease. Recent registry data have indicated a PAH incidence and prevalence of 5.8 and 47.6–54.7 cases per million in adults, respectively (Leber et al., 2021). PAH is associated with a considerable clinical and economic burden (Ogbomo et al., 2022). According to a survey completed in 2021 on the survival status of 461 PAH patients in China, the average annual treatment cost for patients is about two to three times the disposable annual income (Luo et al., 2022). With the low employment rate of PAH patients, the resulting indirect costs are even higher (Fuge et al., 2021).

Combined treatment including targeted drugs is effective for PAH (Hassoun, 2021; Pulmonary Embolism and Pulmonary Vascular Disease Group of Chinese Thoracic Society et al., 2021; Humbert et al., 2022; Ruopp and Cockrill, 2022). In 1995, the first targeted drug, epoprostenol, began a new era of PAH treatment. With the in-depth study of PAH pathogenesis, three signal transduction pathways (the endothelin pathway, NO pathway, and prostacyclin pathway) are involved in PAH occurrence and development (Ruopp and Cockrill, 2022). Subsequently developed targeted drugs, such as endothelin receptor antagonists (ERA), phosphodiesterase 5 inhibitors (PDE5i), soluble guanylate cyclase stimulators (sGCs), prostacyclin analogs, and prostacyclin receptor agonists, also target these three signaling pathways.

Since PAH pathogenesis is unclear, the combination of drugs acting on different signal transduction pathways is a clinical consensus, and the ERA + PDE5i regimen is preferred in China, the United States, and Europe (Klinger et al., 2019; Hassoun, 2021; Pulmonary Embolism and Pulmonary Vascular Disease Group of Chinese Thoracic Society et al., 2021; Humbert et al., 2022; Ruopp and Cockrill, 2022). Nevertheless, in some patients, the disease progresses even after adequate treatment with the combination of ERA and PDE5i. The 2022 ESC/ERS guidelines have recommended that patients with PAH at intermediate-low risk after initial ERA in combination with PDE5i should be considered for sequential addition of selexipag to reduce the risk of clinical worsening. Additionally, sequential intravenous or subcutaneous administration of prostacyclin analogs should be considered in intermediate-high- or high-risk patients with PAH who have received oral therapies or sequential administration of selexipag if adding intravenous or subcutaneous prostacyclin analogs is unfeasible (Humbert et al., 2022).

Selexipag is the first marketed prostacyclin receptor agonist and is currently the only drug acting on the prostacyclin pathway that has been approved by the FDA for oral administration (Panagiotidou et al., 2021). Two randomized controlled trials (RCTs) have compared the efficacy differences in combination therapy to date. GRIPHON, the largest randomized controlled trial of selexipag, has shown that a sequential combination strategy including selexipag reduced the risk of clinical worsening events in patients receiving ERA and PDE5i therapy (hazard ratio [HR] = 0.63; 95% confidence interval [CI] 0.44–0.90) (Coghlan et al., 2018). The TRITON trial has indicated that initial triple oral therapy vs. dual oral therapy might reduce the risk of disease progression (HR = 0.59; 95%CI: 0.32–1.09) (Chin et al., 2021).

However, the combined treatment brings higher costs. Although the Chinese National Centralized Drug Procurement (NCDP) has significantly reduced the costs of ERA and PDE5i, the prices of selexipag and prostacyclin analogs are still high. Based on current drug prices, the average monthly treatment cost of selexipag is $430–2,483 (YaoZh, 2022). That is still a considerable burden for patients and the healthcare system. For the first time, we compared the cost-effectiveness of selexipag added to the combination therapy of ERA and PDE5i from Chinese healthcare system, intending to aid clinicians and decision-makers in the value assessment of this new therapeutic option.

## 2 Methods

### 2.1 Model structure

**FIGURE 1:**
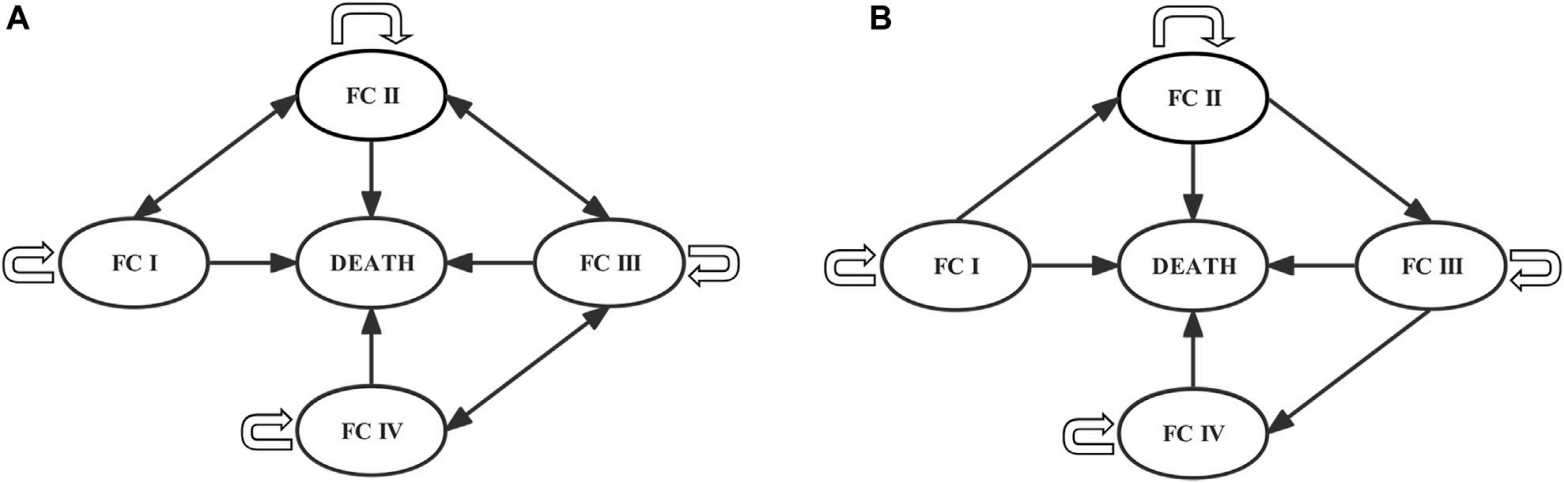
Markov models of the **(A)** first and **(B)** subsequent cycles. Each circle represents a state, lines and arrows represent transition directions between states. Patients can be in only one health state per cycle. FC, functional class.

Markov models were often used to simulate the development and treatment process of chronic diseases over a long period without long-term data from clinical trials. Therefore, we developed a Markov model using TreeAge Pro (Healthcare Version) 2022 (TreeAge Software) to evaluate the long-term effects of PAH treatment in ERA + PDE5i + selexipag and ERA + PDE5i. World Health Organization (WHO) functional class (FC) was derived from an adaptation of the New York Heart Association (NYHA) FC for the assessment of disease severity in patients with PAH. According to the disease progression of PAH, the model was divided into five mutually exclusive Markov states: FC I, FC II, FC III, FC IV, and Death (absorbing state) (Chen et al., 2009; Tran et al., 2015; Coyle et al., 2016). According to the recommendations of the National Institute for Health and Care Excellence (NICE) (Chen et al., 2009) and the Canadian Agency for Drugs and Technologies in Health (CADTH) (Tran et al., 2015), due to the unavailability of data on the long-term effects of therapeutic drugs on disease progression, it is assumed that the patient can maintain the original FC state, improve or deteriorate to the adjacent FC state or enter the death state in the first cycle after receiving targeted drug treatment. In subsequent cycles, treatment could only delay disease progression, and the patients could only remain in the original FC state, deteriorate to an adjacent FC state, or enter the death state (Figure 1).

Parameters required for the model included baseline characteristics of the initial cohort, transition probability, cost, and utility values. Baseline characteristics of the initial cohort were obtained from real-world data in China. Only the TRITON trial reported the subgroup data on the effect on FC of the triple-combination therapy with selexipag for treating PAH compared to the double-combination treatment among the RCTs of selexipag for which trial results have been published. Therefore, clinical efficacy data from the TRITON trial were used to calculate the transition probability of the model between FC states. Cost and utility values were derived from published literature.

The cycle length of the Markov model in the pharmacoeconomic studies on pulmonary arterial hypertension is mainly determined based on the trial length of RCTs. The TRITON trial evaluated the therapeutic effect at week 26. Thus, a 26-week cycle length was chosen for the model in this study. A 30-year time horizon was selected, or the period during which 99% of patients died (Chen et al., 2009; Coyle et al., 2016). We assumed that within the overall time horizon, only patients who entered the death state discontinued medication, and the remaining patients were all receiving drug therapy at all times. The pharmacoeconomic evaluation was conducted in accordance with the Consolidated Health Economic Evaluation Reporting Standards (CHEERS) statement (Husereau et al., 2022a; 2022b). The Supplementary Material provides information on the locations in the main text that correspond to each item in the guidelines.

### 2.2 Patient Population

Based on a real-world study in China (Zhang et al., 2011), the initial cohort was assumed to be 36 years old and contained 75.72% of females and 50% of PAH patients with WHO FC II and III. The initial distribution was 50% for FC II and FC III, respectively.

### 2.3 Clinical inputs

A targeted literature review was conducted to identify appropriate model parameters inputs, as summarized in Table 1. 56.3% of patients with PAH enrolled in the TRITON trial were from North America, and 43.7% were from other regions. The clinical data from the TRITON trial were assumed to be applicable to patients in China. At the time of treatment outcome evaluation at Week 26, of the 22 PAH patients with initial FC II, six patients improved to FC I, and of the 98 PAH patients with initial FC III, 51 patients improved to FC II in the selexipag + macitentan + tadalafil group; only one of the total 123 patients experienced FC deterioration. In the macitentan + tadalafil group, three of 23 PAH patients with initial FC II improved to FC I, and 55 of 95 PAH patients with initial FC III status improved to FC II status; only three of the total 124 patients experienced FC deterioration. Based on the opinions of clinical experts, the acquisition of the probability of improvement in FC was calculated using the number of improvements in each FC subgroup in the TRITON trial. Since the number of deteriorations was minimal for obtaining the deterioration probability of FC, concerning the setting conditions for the economic evaluation of other PAH treatments (Chen et al., 2009; Tran et al., 2015; Coyle et al., 2016), we assumed that the deterioration probability from the overall population applied to each FC state. Because of the short duration of the TRITON trial, it was difficult to obtain an accurate picture of the effects of the treatment on patient mortality. Mortality data from pharmacoeconomic studies on PAH were mainly obtained based on real-world studies (Chen et al., 2009; Tran et al., 2015; Coyle et al., 2016). Therefore, we used the HR of different FC states relative to the general population (Thenappan et al., 2010), combined with the data of the seventh Chinese population census in 2020 (Office of the Leading Group of the State Council for the Seventh National Population Census, 2022), and adjusted according to the age and sex ratios of the initial cohort. The formulas $p=1-e^{-rt}$ and $r=-\frac{1}{t}\;\ln\left(1-p\right)$ were used to calculate the death probability data of each FC state in each cycle, wherein *r* in the formula represented the rate, *p* represented the probability, and *t* was the set time (Fleurence and Hollenbeak, 2007).

**TABLE 1:** Basic parameters input to the model and the ranges of the sensitivity analyses.

Parameter	Base-case value	Lower limit	Upper limit	Distribution	Source
Transition probabilities (per 26 weeks)					
Macitentan + Tadalafil					
Probability of FC improvement					
From FC II to FC I	0.130	0.098	0.163	Beta	Chin et al. (2021)
From FC III to FC II	0.579	0.434	0.724	Beta	Chin et al. (2021)
Probability of FC worsening					
From FC I to FC II	0.024	0.018	0.030	Beta	Chin et al. (2021)
From FC II to FC III	0.024	0.018	0.030	Beta	Chin et al. (2021)
From FC III to FC IV	0.024	0.018	0.030	Beta	Chin et al. (2021)
Selexipag + Macitentan + Tadalafil					
Probability of FC improvement					
From FC II to FC I	0.261	0.196	0.326	Beta	Chin et al. (2021)
From FC III to FC II	0.520	0.390	0.650	Beta	Chin et al. (2021)
Probability of FC worsening					
From FC I to FC II	0.008	0.006	0.010	Beta	Chin et al. (2021)
From FC II to FC III	0.008	0.006	0.010	Beta	Chin et al. (2021)
From FC III to FC IV	0.008	0.006	0.010	Beta	Chin et al. (2021)
Hazard Ratio for Mortality Relative to the General Population by Functional Class					
FC I^a^	5.180	—	—	—	Thenappan et al. (2010)
FC II	22.350	6.860	74.310	Log-normal	Thenappan et al. (2010)
FC III	39.340	12.670	125.040	Log-normal	Thenappan et al. (2010)
FC IV	57.470	18.340	183.430	Log-normal	Thenappan et al. (2010)
Drug costs^b^					
Selexipag					
The first cycle	9,850.780	7,388.085	12,313.475	Gamma	YaoZh (2022)
The ensuing cycle	10,769.930	8,077.448	13,462.412	Gamma	YaoZh (2022)
Macitentan	3,502.980	2,627.235	4,378.725	Gamma	YaoZh (2022)
Tadalafil	480.644	360.483	600.805	Gamma	YaoZh (2022)
Hospitalization costs^b^					
FC I, FC II, FC III	1,290.400	967.800	1,613.000	Gamma	Dufour et al. (2017); Wan et al. (2018); National Bureau of Statistics of China (2023a)
FC IV	2,580.800	1935.599	3,225.999	Gamma	Dufour et al. (2017); Wan et al. (2018); National Bureau of Statistics of China (2023a)
Outpatient registration costs^b^					
	16.874	12.656	21.093	Gamma	Shanghai Municipal Development et al. (2017); Changsha Healthcare Security Administration (2020); Chengdu Healthcare Security Administration and Chengdu Municipal Health Commission (2020); Beijing Municipal Medical Insurance Bureau (2021); Health Commission of Shenyang (2022)
Follow-up costs^b^					
	182.354	136.766	227.943	Gamma	Shanghai Municipal Development et al. (2017); Changsha Healthcare Security Administration (2020); Chengdu Healthcare Security Administration and Chengdu Municipal Health Commission (2020); Beijing Municipal Medical Insurance Bureau (2021); Health Commission of Shenyang (2022)
Utilities (per 26 weeks)					
FC I	0.730	0.640	0.820	Beta	Keogh et al. (2007)
FC II	0.670	0.570	0.770	Beta	Keogh et al. (2007)
FC III	0.600	0.500	0.700	Beta	Keogh et al. (2007)
FC IV	0.520	0.430	0.610	Beta	Keogh et al. (2007)

FC, functional class.

^a^The range of this parameter was not presented in the primary research and without a hypothetical value because the remaining hazard ratios varied widely. Therefore, this variable was not included in the sensitivity analysis.

^b^Cost per 26 weeks. Costs reported in the original literature in RMB, were converted to USD, at the exchange rate on 12 October 2022 (1 USD, 7.17 RMB).

### 2.4 Costs and utilities

From the perspective of the Chinese healthcare system, only direct medical costs were included. Since the administration of selexipag needed to be titrated to a patient-tolerated dose, we used the dosing model of the TRITON trial to calculate the costs of the selexipag dose titration phase and dose maintenance phase, as well as the costs of macitentan and tadalafil. Drug costs were derived from the Chinese open-source Yaozh website (YaoZh, 2022). The median bid price of each province was used for selexipag and macitentan, while the prices after entering the NCDP catalog were used for tadalafil.

According to the Chinese healthcare system, patients must register at the hospital outpatient clinic every 4 weeks for drug prescriptions. One cycle in the model needed to be registered 6.5 times, and the outpatient registration cost was calculated.

The guidelines recommend that patients with PAH should be followed up every 3–6 months to assess their disease risk (Pulmonary Embolism and Pulmonary Vascular Disease Group of Chinese Thoracic Society et al., 2021; Humbert et al., 2022). Follow-up examination items included WHO functional class, blood routine, blood biochemistry, arterial oxygen saturation, brain natriuretic peptide (BNP) or N-terminal (NT)-proBNP, 6-min walk test (6MWT), echocardiography, right heart catheterization (RHC), *etc.* (Wlodarczyk et al., 2016). Outpatient and follow-up examination fees were calculated using the median price of medical services published by the Human Resources and Social Security Bureaus of Shenyang (Health Commission of Shenyang, 2022), Beijing (Beijing Municipal Medical Insurance Bureau, 2021), Chengdu (Chengdu Healthcare Security Administration and Chengdu Municipal Health Commission, 2020), Shanghai (Shanghai Municipal Development et al., 2017), and Changsha (Changsha Healthcare Security Administration, 2020), which represent the geographical area and economic development in China (Liu et al., 2022). Since the cycle length in this model was 26 weeks, combined with the guideline-recommended follow-up period, we assumed that the patients should have a follow-up examination every 26 weeks.

A retrospective study from China has reported the hospitalization costs of patients with PAH (Wan et al., 2018), which we converted to current costs using the Chinese annual consumer price index (healthcare) (National Bureau of Statistics of China, 2023a), and calculated the per-cycle hospitalization costs for each FC status using the monthly hospitalizations of patients with PAH in different FC states (Dufour et al., 2017).

Utility values were derived from a study by Keogh (Keogh et al., 2007), which calculated utility values for different FC states using the SF-36 scale. Quality-adjusted life years (QALYs) were obtained by incorporating the utility value into the model and multiplying it by the life years.

### 2.5 Base-case analysis

After 30 years of long-term simulation, the cumulative costs, QALYs, and incremental cost-effectiveness ratio (ICER) were calculated. The willingness-to-pay threshold (WTP) was $38,223.339 per QALY, which was three times the Chinese *per capita* gross domestic product (GDP) in 2022 (Liu GG et al., 2020; National Bureau of Statistics of China, 2023b; State Administration of Foreign Exchange, 2023). When ICER was less than WTP, triple combination therapy was considered cost-effective. Costs and utilities were analyzed in the base-case analysis using an annual discount rate of 5%, whereas 0% and 8% were used for sensitivity analysis (Liu GG et al., 2020).

### 2.6 Sensitivity analysis

One-way sensitivity and probabilistic sensitivity analyses were conducted to characterize model uncertainty. In one-way sensitivity analysis, the parameters were varied within a 95% CI, with maximum and minimum values reported in the literature (Liu GG et al., 2020). If the range of parameters was unavailable, 25% above and below the values of the base case analysis were assumed. The results of the one-way sensitivity analysis were described using tornado charts. In probability sensitivity analysis, 5,000 sampling iterations were performed using Monte Carlo simulation. The transition probability and utility values adopted beta distribution, the cost adopted gamma distribution, and HR adopted log-normal distribution (Table 1) (Briggs et al., 2012). The analysis results were described using a scatterplot and cost-effectiveness acceptability curve.

### 2.7 Scenario analysis

In the base-case analysis, we used the dosing pattern of the TRITON trial to calculate the selexipag cost. Due to the differences between randomized controlled trials (RCTs) and clinical practice in the mode of administration, we assumed that patients would use selexipag for scenario analysis at the lowest daily dose (0.2 mg, bid) and the highest daily dose (1.6 mg, bid). Furthermore, we performed a scenario analysis of different FC percentages of PAH patients in the initial cohort. We assumed that all initial cohorts were in FC II or FC III state to explore the cost-effective of two intervention options for patients with PAH with different disease severity.

## 3 Results

### 3.1 Base-case analysis

Selexipag + macitentan + tadalafil regimen cost more than macitentan + tadalafil ($357,807.5884 vs. $116,534.543, respectively) and was more effective (7.234 QALYs vs. 6.666 QALYs, respectively) (Table 2). The ICER was $424,746.070 per QALY, which was higher than the preset WTP ($38,223.339 per QALY).

**TABLE 2:** Summary of base-case analyses.

Strategy	Cost	Incremental cost	QALYs	Incremental QALYs	ICER ($/QALY)
Macitentan + Tadalafil	$116,534.543	——	6.666	——	——
Selexipag + Macitentan + Tadalafil	$357,807.588	$241,273.044	7.234	0.568	424,746.070

ICER, incremental cost-effectiveness ratio; QALY, quality-adjusted life year.

### 3.2 One-way sensitivity analysis

**FIGURE 2:**
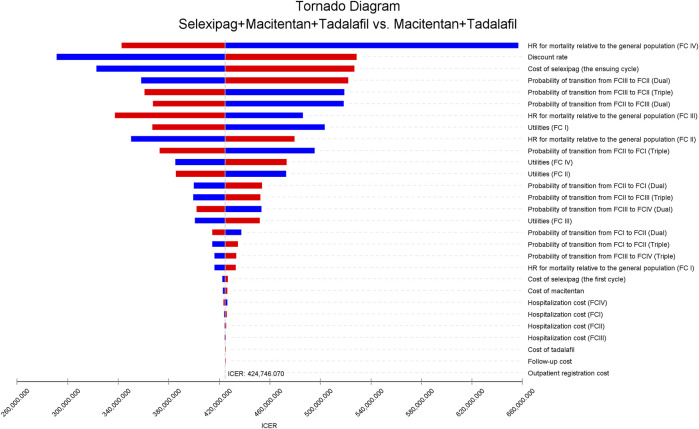
The tornado diagram of univariable sensitivity analyses shows the effect on ICER as each variable varies within its range. The vertical gray line represents the value of ICER in the base-case analysis. The blue bar indicates that the ICER increases as parameter values decrease, and the red bar represents that the ICER decreases as parameter values increase. Dual, macitentan + tadalafil; triple, selexipag + macitentan + tadalafil; FC, functional class; HR, hazard ratio; ICER, incremental cost-effectiveness ratio.

Model results were most sensitive to the HR for mortality of patients with FC IV relative to the general population, discount rate, and the cost of selexipag. When the parameters changed within the preset range, ICER was always higher than WTP (Figure 2). We further increased the upper and lower limits of parameters and explore the impact on the cost-effectiveness of reducing the cost of selexipag with other parameters unchanged. The combination of selexipag with macitentan plus tadalafil was cost-effective when the monthly cost of selexipag was less than $ 68.66. The mean monthly treatment cost for the minimum and maximum doses of selexipag ranged from $430 to $2,483. As a result, the three-drug combination exhibited cost-effectiveness only when the price of selexipag was reduced by 84.03% and 97.23% for the lowest and highest doses, respectively.

### 3.3 Probabilistic sensitivity analysis

**FIGURE 3:**
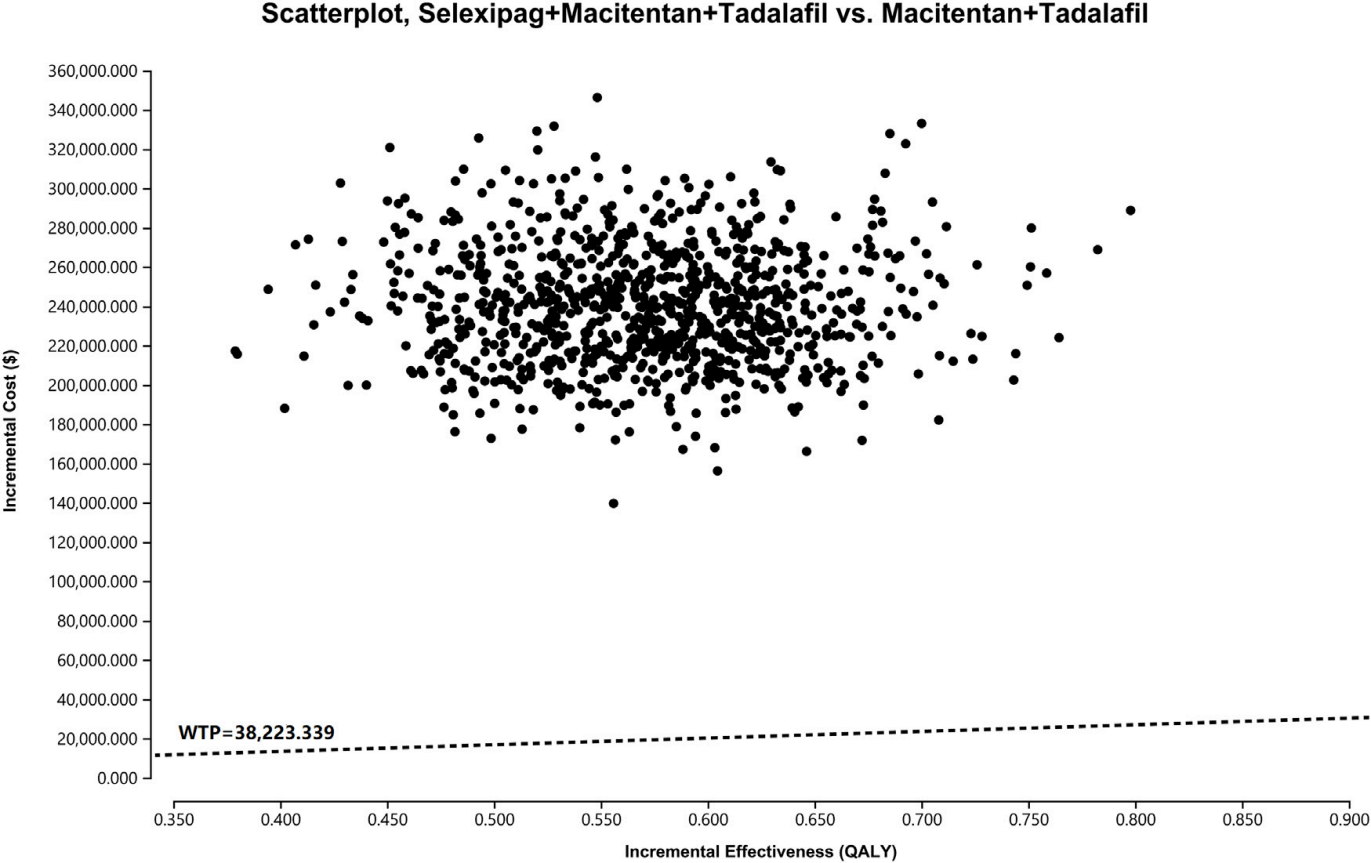
The scatterplot depicts the results of the Monte Carlo analysis. The black dots show 5,000 iterations, and the dashed line indicates the preset WTP. WTP, willingness-to-pay.

**FIGURE 4:**
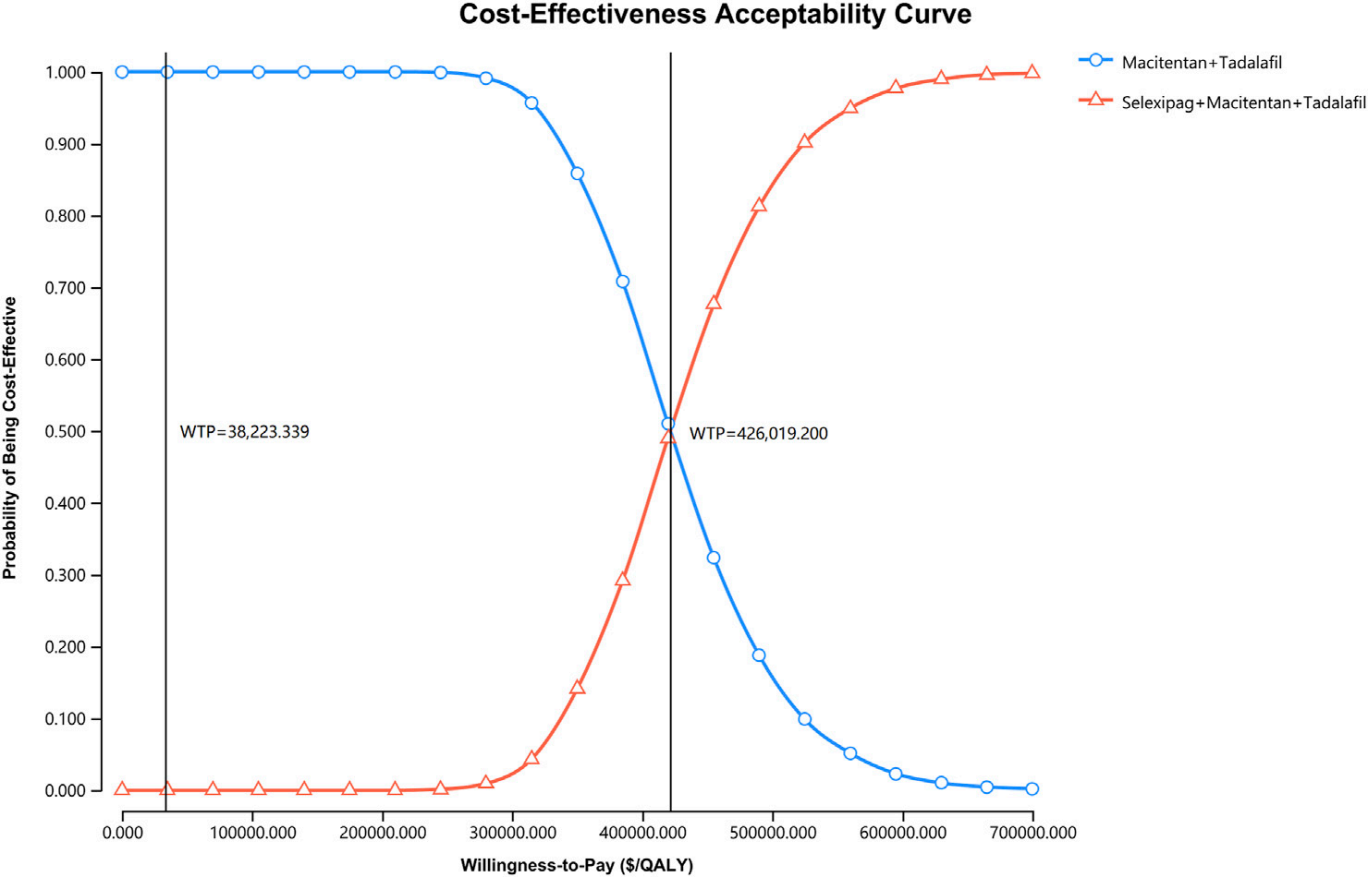
The cost-effectiveness acceptability curve indicates the probability of cost-effectiveness at different WTP thresholds based on the uncertainty of the parameters after 5,000 Monte Carlo simulations. The line consisting of circles demonstrates the strategy of macitentan + tadalafil, and the line consisting of triangles shows the strategy of selexipag + macitentan + tadalafil. The vertical dash lines indicate various WTP values. QALY, quality-adjusted life year; WTP, willingness-to-pay.

Probabilistic sensitivity analysis showed that the probability of cost-effectiveness for the macitentan + tadalafil + selexipag regimen was 0% when the WTP was $38,223.339 per QALY (Figure 3), and the probability was greater than 50% for the triple combination therapy only if the WTP was more significant than $426,019.200 per QALY (Figure 4).

### 3.4 Scenario analysis

When patients were given selexipag at the lowest daily dose of 0.2 mg bid, selexipag + macitentan + tadalafil led to 0.568 more QALYs compared to macitentan + tadalafil at an increased cost of $65,834.378. The ICER was $115,897.296 per QALY. When patients were given selexipag at the highest daily dose of 1.6 mg bid, the ICER was $628,896.969 per QALY. In both cases, the ICER was higher than the preset WTP.

We assumed that all patients with PAH were in FC II state at the beginning of the model simulation, the ICER was $301,750.260 per QALY. When all patients with PAH were in FC III state in the initial cohort, the ICER was $820,274.639 per QALY. In both cases, the ICER was higher than the preset WTP. Therefore, compared with the macitentan + tadalafil, the selexipag + macitentan + tadalafil regimen was not cost-effective for PAH in any of the above scenarios.

## 4 Discussion

Since PAH is a progressive disease that requires lifelong targeted drug treatment, the AMBITION trial (Galiè et al., 2015), GRIPHON trial (Sitbon et al., 2015), and TRITON trial (Chin et al., 2021) have demonstrated the value of targeted drug combined treatment for PAH. In the AMBITION trial, initial combination therapy with ambrisentan and tadalafil resulted in a significantly lower risk of clinical failure events than the risk with ambrisentan or tadalafil monotherapy among participants with PAH who had not received previous treatment. GRIPHON and TRITON trials provided sufficient evidence for combined treatment including selexipag in patients receiving ERA and PDE5i combination therapy (Pulmonary Embolism and Pulmonary Vascular Disease Group of Chinese Thoracic Society et al., 2021; Humbert et al., 2022).

China, as the most populous country in the world, offers a unique way to address the PAH burden. Some targeted drugs, such as bosentan, ambrisentan, macitentan, and selexipag, are listed in the National Reimbursement Drug List. Since 2019, the Chinese government has implemented a policy of centralized procurement of drugs with involved ambrisentan, sildenafil, and tadalafil. These policies have made a big difference in reducing the PAH burden, but it is still not enough. According to a survey in China, approximately 71% of patients with PAH spend 79.8% of their family income on disease treatment, and more than 50% of patients do not use any targeted drugs due to unaffordable costs (Zhai et al., 2017). The inability to work due to the deterioration of cardiac function in PAH patients will lead to a low employment rate and greater indirect costs (Howard et al., 2012; Matura et al., 2014). Therefore, it is necessary to conduct a comprehensive economic evaluation of targeted drugs for PAH not only limited to ERA and PDE5i but also sGCs, prostacyclin analogs, and prostacyclin receptor agonists to provide a more comprehensive reference for clinical decision- and policy-making.

To the best of our knowledge, this study was the first cost-effectiveness analysis of selexipag for PAH from the perspective of the healthcare system in China. Markov model was constructed according to different WHO FC states to simulate the natural process of PAH. The cost parameters were all from the Chinese healthcare system, and other parameters were obtained from published literature and public databases. We compared the cost-effectiveness of ERA + PDE5i + selexipag and ERA + PDE5i based on the latest clinical guideline recommendations and the actual treatment situation of PAH in China. The choice of macitentan and tadalafil as the representative drug of ERA and PDE5i was based on the TRITON trial (Chin et al., 2021). Meanwhile, ambrisentan, another ERA, is a government-centralized procurement drug with a lower treatment cost than macitentan. Ambrisentan, macitentan, and tadalafil are most commonly used in the initial treatment of PAH in China. Therefore, the results of this study are generalizable. This study showed that sequential selexipag might not be cost-effective for patients with PAH who failed to control their condition after combined treatment of ERA and PDE5i when the WTP was 38,223.339 per QALY. One-way sensitivity and probability sensitivity analyses confirmed the conclusion.

Four studies on the pharmacoeconomics of selexipag have been conducted in Canada (Pharmacoeconomic Review Report: Selexipag Uptravi, 2017), Sweden (Wlodarczyk et al., 2016), and Greece (Solakidi et al., 2017a; 2017b). The last three studies have not been able to obtain the full text or specify research details. A study from Sweden has compared the cost-effectiveness of sequential administration of selexipag and inhaled iloprost for PAH patients who had received ERA and PDE5i from the societal perspective. Selexipag was more cost-effective than inhaled iloprost (ICER 37,350 SEK per QALY) (Wlodarczyk et al., 2016). A Greece study has compared the cost-effectiveness of sequential administration of selexipag and subcutaneous treprostinil in PAH patients with FC III who had received ERA and PDE5i but had insufficient efficacy from a Greek payer perspective. Selexipag was less costly and more effective than subcutaneous treprostinil, thus, making it to be a dominant strategy (Solakidi et al., 2017a). Based on the previous study, the Greek research group conducted a budget impact analysis to treat PAH with selexipag for a 5-year time horizon (2017–2021). The result showed that using a triple combination therapy containing selexipag in patients with PAH FC III could save the public payers’ budget in the Greek healthcare setting (Solakidi et al., 2017b). A study from Canada has compared the cost-effectiveness of adding selexipag to background therapy (ERA and/or PDE5i, or no intervention) using GRIPHON trial efficacy data (Sitbon et al., 2015) from a third-party payer’s perspective and calculated the ICER to be $485,000 per QALY, which was higher than Canadian 2015 WTP ($50,000 per QALY). Hence, adding selexipag to background therapy would not be cost-effective (Pharmacoeconomic Review Report: Selexipag Uptravi, 2017). However, this study did not report ICER calculated with the sequential addition of selexipag when background treatment was ERA + PDE5i. There are differences between our research and other studies in terms of research perspective, methods, and comparative treatment regimes. Our analysis is the first pharmacoeconomic assessment for evaluating the cost and effectiveness of treating PAH with selexipag added to the combination of ERA and PDE5i.

This study was subject to several limitations. First, it was possible to underestimate the therapeutic effect on the assumption that FC improvement was only present in the first cycle. Although other studies hypothesized that treatment improvement existed in the entire simulation duration range (Thongsri et al., 2016), we still assumed that FC improvement only existed in the first cycle due to the lack of long-term clinical data (Chen et al., 2009; Tran et al., 2015; Coyle et al., 2016). Second, most current pharmacoeconomic studies on PAH treatment drugs set the initial cohort age at 50 years (Chen et al., 2009; Coyle et al., 2016). According to the data from real-world research in China (Zhang et al., 2011), this study set the initial cohort age at 36 years. Bias might have been present due to the epidemiological data (Zhang et al., 2011) with PAH in China published in 2011 without update for 10 years. Third, in the TRITON trial, the number of FC worsening in macitentan + tadalafil and macitentan + tadalafil + selexipag at week 26 was three and one, respectively. Hence, the transfer probabilities calculated based on these small sample data might have been biased. A small number of cases is a common problem in exploring rare diseases. Accordingly, we retrieved published literature and public databases as much as possible. There have been only three RCTs studying selexipag at present (Simonneau et al., 2012; Sitbon et al., 2015; Chin et al., 2021), and the other two (Simonneau et al., 2012; Sitbon et al., 2015), except for the TRITON trial, have yet to report the specific effectiveness data of the double combination therapy subgroup. The demographic characteristics of the initial cohort of the model were derived from real-world data from China, which differed from the baseline characteristics of the TRITON trial. However, we controlled for uncertainty in the outcome by performing sensitivity and scenario analyses. This limitation needs to be improved in combination with subsequent published clinical trials. Fourth, with reference to the economic evaluation assumptions of other oral treatments for PAH (Tran et al., 2015; Coyle et al., 2016; Thongsri et al., 2016), adverse events due to oral PAH therapies were generally minor. This study did not consider the adverse reaction management cost incurred from oral administration. Consequently, the overall treatment cost might have been underestimated. A further collection of management costs of adverse events is needed to refine our findings in the future. Fifth, the lack of clinical trial data makes it currently difficult to compare the cost-effectiveness of selexipag with other targeted drugs. If appropriate clinical trials or real-world research data become available, information on the cost-effectiveness of selexipag compared to other drugs used in clinical practice will be accessed to determine its appropriate position in the treatment pathway based on cost-effectiveness. Finally, due to the limited availability of long-term efficacy data for selexipag in treating PAH, further research is needed to collect longer-term data that can be applied in pharmacoeconomic studies.

## 5 Conclusion

As the only oral drug acting on the prostacyclin pathway, selexipag represents a significant breakthrough and novel addition to the available combined treatment options for PAH. Adding selexipag to the combined treatment of ERA and PDE5i was not cost-effective at a WTP threshold set as three times Chinese GDP *per capita* in 2022, from the perspective of the healthcare system in China. HR for mortality of patients with FC IV relative to the general population, discount rate, and the cost of selexipag appeared to be the primary drivers of ICER. Novel pricing strategies may mitigate high drug costs to make treatment options, which include selexipag, cost-effective.

## Data Availability

The original contributions presented in the study are included in the article/Supplementary Material, further inquiries can be directed to the corresponding authors.
